# The Chromosome 9p21 Variant Not Predicting Long-Term Cardiovascular Mortality in Chinese with Established Coronary Artery Disease: An Eleven-Year Follow-Up Study

**DOI:** 10.1155/2014/626907

**Published:** 2014-04-02

**Authors:** I-Te Lee, Mark O. Goodarzi, Wen-Jane Lee, Jerome I. Rotter, Yii-der Ida Chen, Kae-Woei Liang, Wen-Lieng Lee, Wayne H.-H. Sheu

**Affiliations:** ^1^Division of Endocrinology and Metabolism, Department of Internal Medicine, Taichung Veterans General Hospital, Taichung 40705, Taiwan; ^2^School of Medicine, Chung Shan Medical University, Taichung 40201, Taiwan; ^3^School of Medicine, National Yang-Ming University, Taipei 11221, Taiwan; ^4^Division of Endocrinology, Diabetes and Metabolism, Cedars-Sinai Medical Center, Los Angeles, CA 90048, USA; ^5^Medical Genetics Institute, Cedars-Sinai Medical Center, Los Angeles, CA 90048, USA; ^6^Department of Medical Research, Taichung Veterans General Hospital, Taichung 40705, Taiwan; ^7^Department of Ob/Gyn, Cedars-Sinai Medical Center, Los Angeles, CA 90048, USA; ^8^Cardiovascular Center, Taichung Veterans General Hospital, Taichung 40705, Taiwan

## Abstract

*Introduction*. We examined whether the variant at chromosome 9p21, rs4977574, was associated with long-term cardiovascular mortality in Han Chinese patients with coronary artery disease (CAD). *Methodology*. Subjects who underwent coronary angiography for chest pain were consecutively enrolled. Fasting blood samples were collected for laboratory and genotype assessments. The information was correlated with data collected from the national death database. *Results*. There were 925 cases with CAD and 634 without CAD enrolled in the present study. The G allele conferred a significant increase in risk of CAD (odds ratio = 1.47, *P* = 0.003 in the dominant model; odds ratio = 1.36, *P* = 0.018 in the recessive model). During a median of 11 years (inter-quartile range between 5.2 and 12.5 years) of follow-up, neither the total nor the cardiovascular mortality was different among CAD subjects with different genotypes. Using Cox regression analysis, genotypes of rs4977574 still failed to predict cardiovascular mortality (hazard ratio = 1.25, *P* = 0.138 in the dominant model; hazard ratio = 1.05, *P* = 0.729 in the recessive model). *Conclusions*. The rs4977574 at chromosome 9p21 is associated with presence of CAD in Han Chinese. However, rs4977574 could not predict cardiovascular mortality in these CAD subjects during the eleven-year period of the study.

## 1. Introduction

Coronary artery disease (CAD) is the leading cause of death worldwide [1]. Heredity plays an important role in CAD susceptibility [2]. The chromosome 9p21 region has been shown to be associated with CAD, and several single nucleotide polymorphisms (SNPs) selected from Caucasians have been replicated in Chinese [3–5]. However, SNPs in chromosome 9p21 contribute diverse intensities of risk to cardiovascular disease, and it has been reported that the effect size of identified SNPs is dependent on the chosen population [6–8].

Among the tag SNPs, rs4977574 has been initially demonstrated to be associated with risk of CAD in the Myocardial Infarction Genetics Consortium (MIGen) study [9]. The association between rs4977574 and CAD has also been found in several populations [10–13]. However, a small study comprising 334 subjects did not show any significant association between CAD risk and rs4977574 in a Chinese population [14].

Although rs4977574 was recently reported to be associated with cardiovascular incidence in the subjects without CAD at baseline [15], results from longitudinal assessments between 9p21 variants and cardiovascular mortality in subjects with established CAD were inconsistent. In the Global Registry of Acute Coronary Events (GRACE) genetic study, the 9p21 variant showed a significant association with cardiovascular death [16]. However, Virani et al. [17] recently reported that the 9p21 variant was not associated with cardiovascular outcome. In order to clarify the long-term impact of 9p21 variant on cardiovascular mortality in CAD patients, we therefore examined the polymorphism of rs4977574 in a Taiwanese cohort of Han Chinese descent in an eleven-year follow-up duration.

## 2. Material and Methods

### 2.1. Patients

The participants were enrolled during hospitalization in the Cardiovascular Department in Taichung Veterans General Hospital between March 1999 and March 2000. The study comprised both case-control and follow-up assessments for subjects undergoing coronary angiography due to angina and clinical suspicion of ischemic heart disease. Blood samples were collected after the subjects had fasted overnight. Mortality data up to 2011 were collected for the follow-up assessment. The study protocol was approved by the Institutional Review Board of Taichung Veterans General Hospital, Taichung, Taiwan.

### 2.2. Methods

Genomic DNA was extracted from patients' peripheral blood leukocytes using a QIAamp DNA Blood Mini Kit (Qiagen, Hilden, Germany). Genotypes of rs4977574 were determined using TaqMan allelic discrimination assay (Custom TaqMan SNP Genotyping Assay). The genotyping reaction was amplified and detected on a StepOnePlus Real-Time PCR (Applied Biosystems, Foster City, USA).

Plasma glucose was determined using the glucose oxidase-peroxidase method (Wako Diagnostics, Tokyo, Japan). Serum cholesterol and triglyceride levels were determined by the enzymatic method using commercial kits (Wako Diagnostics, Tokyo, Japan). High-density lipoprotein (HDL) cholesterol was determined after the precipitation of apo B-containing lipoproteins. Low-density lipoprotein (LDL) cholesterol concentration was calculated using the formula of Friedewald et al. [18] if fasting serum triglyceride was less than 4.5 mmol/L. Otherwise, LDL cholesterol was determined after separation of very-low-density lipoprotein (VLDL) from serum by ultracentrifugation and precipitation of apo B-containing particles.

### 2.3. Exposure Information

The subjects were assigned to the CAD group if they fulfilled one or both of the following criteria: (1) a history of myocardial infarction or coronary revascularization or (2) 50% or more luminal narrowing in any coronary artery according to angiography. Subjects who did not fulfill any of these criteria were assigned to the non-CAD group. Diabetes mellitus was defined as (1) a history of diabetes mellitus or use of antidiabetic medications or (2) fasting blood glucose levels equal to or higher than 7.0 mmol/L. Hypertension was defined as (1) a history of hypertension or use of antihypertensive medications or (2) systolic blood pressure higher than 140 mmHg or diastolic blood pressure higher than 90 mmHg.

The coronary artery lesions were assessed at the angiography viewing workstation using software for quantitative analysis (Philips Inturis Suite, R2.2, Philips Medical Systems, Eindhoven, Netherlands). The baseline CAD severity was evaluated by Gensini score, which was calculated by the sum of obstructive severity score times the lesion location score system [19].

The mortality data were provided by the Collaboration Center of Health Information Application (CCHIA), Department of Health, Executive Yuan, Taiwan. The causes of death were determined according to the International Classification of Disease, 9th Revision, Clinical Modification (ICD-9 CM) diagnostic criteria. Cardiovascular disease was defined as CAD (402, 404, 410, 411, 412, 413, 414, 426, 427, 428, and 429), cerebrovascular disease (430, 431, 432, 433, 434, 435, 436, 437, and 785.9), and peripheral artery disease (440.2, 443.8, 443.9, and 444.2) [20].

### 2.4. Statistical Analysis

Data are presented as mean ± standard deviation (SD). The genotype distribution was tested for Hardy-Weinberg equilibrium by the goodness-of-fit test. Chi-square test was used to assess the differences in gender, genotypes, diabetes, hypertension, and smoking status. The differences in continuous variables at baseline were analyzed by independent *t*-test. Multivariate logistic regression analyses were used to assess the genetic effects on CAD at baseline. Multivariable Cox proportional-hazard regression analyses were used to examine the cardiovascular mortality between genotypes. Statistical analysis was performed using SPSS 19.0 (SPSS Inc., Chicago, IL, USA).

## 3. Results

### 3.1. Case-Control Assessments at Baseline

**Figure 1:**
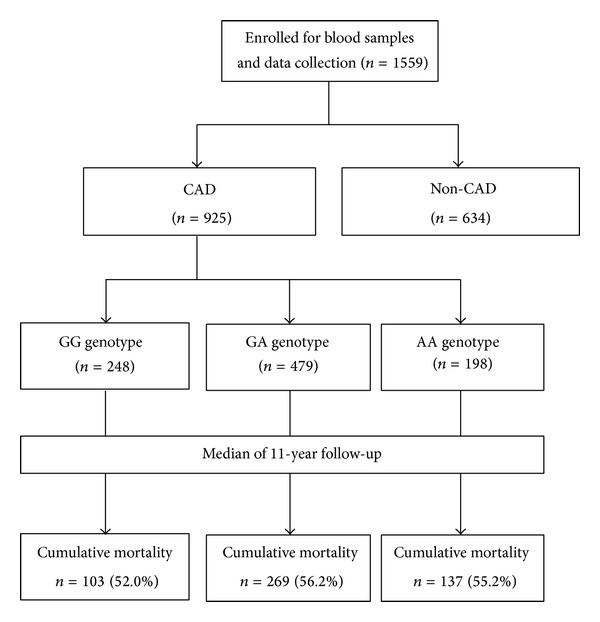
Flow diagram of enrollment of study subjects.

A total of 1559 subjects were enrolled with 925 in the CAD group and 634 in the non-CAD group (Figure 1). The distribution of alleles was in agreement with the Hardy-Weinberg equilibrium. The clinical features of the two study groups are shown in Table 1. The CAD group was significantly older with male dominance compared with the non-CAD group (both *P* values less than 0.001). There were significantly higher proportions of current smoking status (*P* < 0.001), hypertension (*P* = 0.032), and diabetes mellitus (*P* < 0.001) in the CAD group than those in the non-CAD group. Fasting serum concentrations of LDL cholesterol and total cholesterol were also significantly higher in the CAD group than those in the non-CAD group (both *P* values less than 0.001). The genotype distributions showed that G allele of rs4977574 was overrepresented in the CAD group compared to the non-CAD group (*P* = 0.002 for genotype distribution and *P* = 0.001 for allele distribution, resp.). The risk allele remained independently associated with CAD after adjusting for age, gender, smoking, hypertension, diabetes, and LDL cholesterol in the logistic regression model (Table 2).

### 3.2. Longitudinal Cardiovascular and Total Mortality for CAD Patients

**Figure 2:**
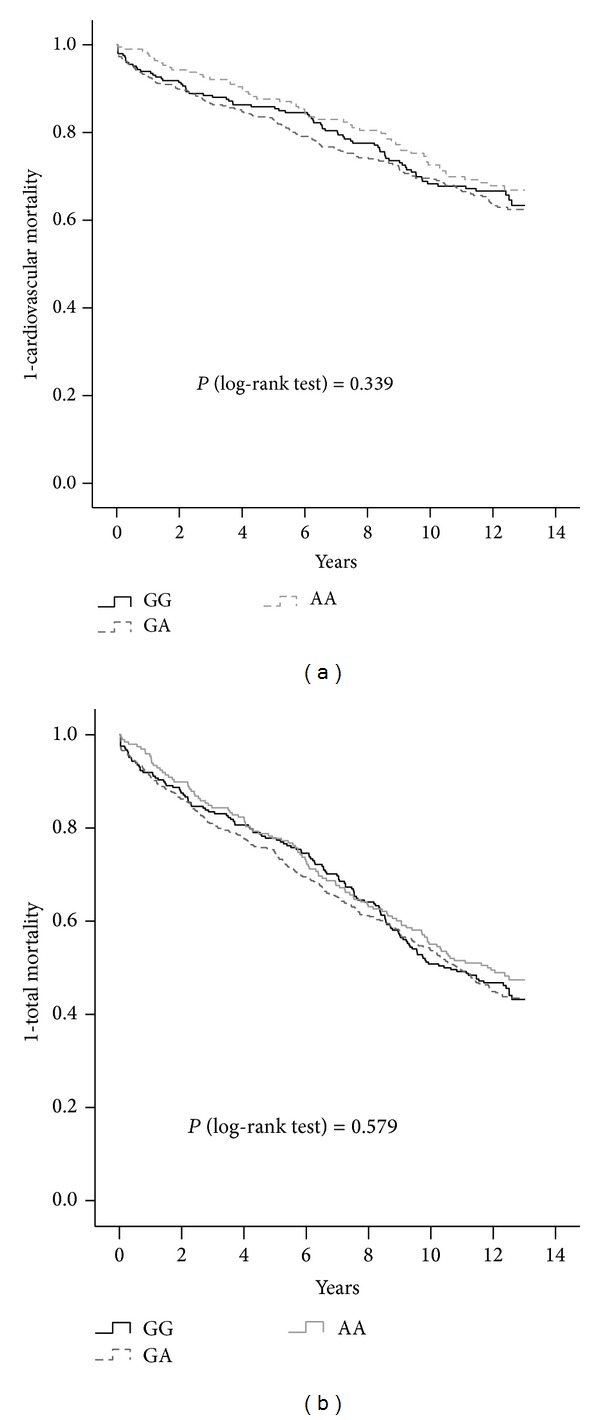
Kaplan-Meier curves showing (a) proportion without cardiovascular mortality and (b) proportion without total mortality based on different genotypes in the patients with CAD.

Among the 925 patients with CAD at baseline, there were 248 with GG genotype, 479 with GA genotype, and 198 with AA genotype (Table 3). The CAD severity, based on the Gensini score, was not significantly different among all genotypes (*P* = 0.412). During the follow-up period (median of 11.0 years, interquartile range between 5.2 and 12.5 years), there was no significant difference in cardiovascular mortality or total mortality among the CAD patients with different genotypes (Figure 2). Using Cox regression analysis, the risk G allele was not associated with cardiovascular mortality and total mortality in either the dominant model or the recessive model after adjustment for age, gender, smoking status, past history of myocardial infarction, statins treatment, presence of hypertension, and presence of diabetes (Table 4).

## 4. Discussion

Our major findings were that the G allele of SNP rs4977574 was associated with the presence of CAD but failed to predict cardiovascular death in the Han Taiwanese patients with established CAD. The case-control findings of our study are in-line with results reported in other populations [9–13]. SNP rs4977574 is located at a locus with a gene encoding a large antisense noncoding RNA (ANRIL) [21, 22]. ANRIL might be involved in atherosclerosis by being expressed in the endothelial cells and monocyte-derived cells [21, 23]. Upstream from the locus, the cyclin-dependent kinase inhibitor 2A (CDKN2A) and 2B (CDKN2B) genes are associated with transcriptional regulation of ANRIL [24–26]. It is also important to note that the baseline Gensini scores were not significantly different among the CAD patients with three different genotypes. In accordance with our observations, Chen et al. [27] reported that the 9p21 variant is not associated with CAD severity in subjects with established CAD. Similar findings, reported in Chinese subjects with myocardial infarction, showed that severity of coronary lesion was not significantly different among different genotypes of the 9p21 variant [28]. Further investigations are warranted to clarify the underlying pathogenesis contributing to the observed association between chromosome 9p21 variants and coronary lesions.

In the present study, rs4977574 failed to predict cardiovascular or total mortality in subjects with CAD during the eleven-year period of follow-up. Our finding in CAD Chinese was different from results in subjects without CAD [15]. Furthermore, the GRACE genetic study showed the 9p21 variant was significantly associated with recurrent myocardial infarction within only six months [16], but Peng et al. [28] reported that the 9p21 variant could not predict total mortality in subjects following myocardial infarction in a 2.5-year study. Virani et al. [17] also found no significant association between the 9p21 variant and cardiac death in subjects with established CAD in a 3.2-year follow-up. One of the main strengths of the present study was that long-term mortality in a follow-up period of 11 years was analyzed in CAD patients, using death records as the endpoint to test our hypothesis.

It is well acknowledged that the current established risk factors of atherosclerosis are not able to fully account for cardiovascular death [29]. The discrepancy probably results from different pathophysiological mechanisms. Endothelial dysfunction and monocyte activation play roles in inflammation and subsequent development of coronary lesions [30, 31]. However, the atherosclerotic biomarkers and monocyte activity were poor predictors of longitudinal cardiovascular events in patients with established CAD [32]. In fact, cardiovascular death might be associated with the critical mechanisms of plaque stabilization and arrhythmia in CAD patients [33–35]. In large-scale genome-wide association studies (GWAS), susceptibility variants were determined based on case-control studies, and thus it might not be possible to prospectively predict the outcomes of diseases [36]. Previous authors have asserted that the effects of the 9p21 genetic polymorphisms might be only associated with coronary lesion but not capable of predicting cardiovascular mortality [37, 38]. Furthermore, cardiovascular death might be associated with many common diseases, which in turn may be linked to both genetic and environmental factors [39, 40]. The association between chromosome 9p21 variants and sequential cardiovascular mortality might be abated by the medical treatment or procedural intervention during the follow-up period [41]. Along the same lines, it has been reported that the 9p21 variants could not predict the cardiovascular outcomes in subjects with coronary lesions after drug-eluting stent placement [42].

Cavender et al. [43] reported a total mortality of 37% in subjects with CAD at five years. In the present study, the accumulative total mortality of about 55% was observed in a median 11-year period of follow-up. Ethnic differences in mortality were reported in CAD subjects, with a higher mortality in Asians compared with that in Caucasians [44, 45]. However, the prognosis of CAD seems to be better in Chinese than that in south Asians, although the mechanism is not well understood [46]. In a recent report from a Vienna cohort study with a similar duration of follow-up [47], a lower total mortality rate (39%) in the CAD subjects was observed, although the proportion of cardiovascular cause (57%) was similar to that in the present study (56.6%). Because the majority of deaths in the present study were cardiovascular causes, the total mortality findings were close to those of cardiovascular mortality.

There were some limitations in the current study. First, the cardiovascular mortality data was collected from the national mortality registry which covers 99.6% of the total population in Taiwan [48]. However, nonfatal cardiovascular events could not be traced in the follow-up assessment as these data are not provided in the database. Second, we assessed cardiovascular mortality rather than CAD mortality due to indistinct classifications of death causes; for example, some types of CAD might be classified to cardiovascular disease (ex. ICD-9 code of 429.2) [49]. Furthermore, we could not collect information on the effect of environmental factors and treatments during the follow-up period.

In conclusion, the SNP rs4977574 at chromosome 9p21 was an independent risk factor for CAD in Taiwanese individuals of Han Chinese descent. However, this SNP could not predict the total or cardiovascular mortality in these subjects, who had CAD at baseline, during the eleven-year period of follow-up.

## Figures and Tables

**Table 1:** The clinical characteristics and genotypic frequency of study subjects at baseline.

	CAD (*N* = 925)	Non-CAD (*N* = 634)	*P*
Age (year)	67.6 ± 10.0	64.5 ± 10.8	<0.001
Male, *n* (%)	742 (80.2%)	408 (64.4%)	<0.001
BMI (kg/m^2^)	25.3 ± 3.3	25.3 ± 3.5	0.954
Systolic BP (mmHg)	126 ± 13	126 ± 11	0.490
Diastolic BP (mmHg)	71 ± 10	71 ± 11	0.813
Current smoking, *n* (%)	260 (28.1%)	128 (20.2%)	<0.001
Hypertension, *n* (%)	466 (50.4%)	280 (44.2%)	0.032
Diabetes mellitus, *n* (%)	344 (37.2%)	137 (21.6%)	<0.001
Triglyceride (mmol/L)	1.6 ± 1.3	1.5 ± 1.4	0.045
Total cholesterol (mmol/L)	4.8 ± 1.2	4.4 ± 1.2	<0.001
HDL cholesterol (mmol/L)	1.1 ± 0.3	1.2 ± 0.4	0.312
LDL cholesterol (mmol/L)	2.9 ± 1.0	2.6 ± 0.9	<0.001
Fasting glucose (mmol/L)	6.8 ± 2.7	6.1 ± 2.1	<0.001
Genotype (rs4977574)			0.002
AA	198 (21.4%)	181 (28.5%)	
AG	479 (51.8%)	318 (50.2%)	
GG	248 (26.8%)	135 (21.3%)	
Allele (rs4977574)			*P* = 0.001
A	875 (47.3%)	680 (53.6%)	
G	975 (52.7%)	588 (46.4%)	

BMI: body mass index, BP: blood pressure, HDL: high-density lipoprotein, and LDL: low-density lipoprotein.

**Table 2:** Multivariate association between the rs4977574 genotypes and presence of coronary artery disease (CAD).

	Dominant model*		Recessive model*	
	Odds ratio (95% CI)	*P*	Odds ratio (95% CI)	*P*
Crude	1.47 (1.16, 1.85)	0.001	1.35 (1.07, 1.72)	0.013
Adjusted^1^	1.47 (1.16, 1.86)	0.002	1.41 (1.10, 1.81)	0.006
Adjusted^2^	1.47 (1.14, 1.89)	0.003	1.36 (1.05, 1.77)	0.018

*Risk allele: G.

^1^For age and gender.

^2^For age, gender, smoking, hypertension, diabetes, and LDL cholesterol.

**Table 3:** Baseline characteristics of the subjects with coronary artery disease (CAD) according to the rs4977574 genotypes.

	GG (*N* = 248)	GA (*N* = 479)	AA (*N* = 198)	*P*
Age (year)	66 ± 10^a^	68 ± 10^b^	68 ± 10^b^	0.009
Male, *n* (%)	193 (77.8%)	385 (80.4%)	164 (82.8%)	0.416
BMI (kg/m^2^)	25.7 ± 3.4	25.1 ± 3.3	25.2 ± 3.3	0.191
Systolic BP (mmHg)	127 ± 12	125 ± 13	127 ± 13	0.062
Diastolic BP (mmHg)	72 ± 10	71 ± 10	70 ± 10	0.264
Current smoking, *n* (%)	75 (30.2%)	132 (27.6%)	53 (26.8%)	0.668
Hypertension, *n* (%)	170 (68.5%)	297 (62.0%)	127 (64.1%)	0.218
Diabetes mellitus, *n* (%)	126 (50.8%)	228 (47.6%)	92 (46.5%)	0.612
Previous MI	26 (10.5%)	45 (9.4%)	21 (10.6%)	0.844
Statins treatment	11 (4.4%)	35 (7.3%)	15 (7.6%)	0.275
Triglyceride (mmol/L)	1.7 ± 1.4	1.5 ± 1.0	1.6 ± 1.6	0.237
Total cholesterol (mmol/L)	4.7 ± 1.2	4.7 ± 1.1	4.8 ± 1.3	0.520
HDL cholesterol (mmol/L)	1.1 ± 0.3	1.2 ± 0.3	1.1 ± 0.3	0.198
LDL cholesterol (mmol/L)	2.8 ± 0.9	2.9 ± 1.0	3.0 ± 1.1	0.275
Fasting glucose (mmol/L)	6.8 ± 2.7	6.9 ± 2.7	6.7 ± 2.7	0.780
Gensini score*	21.5 (8.0, 48.8)	20.0 (8.0, 48.0)	20.0 (6.4, 41.3)	0.412

^a,b^Significant difference between a and b.

*Data of Gensini score presented as median (interquartile range).

BMI: body mass index, BP: blood pressure, HDL: high-density lipoprotein, LDL: low-density lipoprotein, and MI: myocardial infarction.

**Table:** (a) Cardiovascular mortality

	Dominant model			Recessive model		
	HR	(95% CI)	*P*	HR	(95% CI)	*P*
Age (10 years)	1.80	(1.55, 2.09)	<0.001	1.79	(1.54, 2.08)	<0.001
Male	1.05	(0.78, 1.41)	0.741	1.05	(0.78, 1.41)	0.755
Current smoking	0.90	(0.68, 1.20)	0.474	0.91	(0.68, 1.20)	0.498
Hypertension	0.91	(0.71, 1.160)	0.456	0.91	(0.71, 1.16)	0.444
Diabetes mellitus	1.84	(1.46, 2.33)	<0.001	1.84	(1.46, 2.34)	<0.001
Previous MI	1.20	(0.83, 1.72)	0.336	1.20	(0.84, 1.74)	0.318
Statins treatment	0.75	(0.44, 1.28)	0.285	0.75	(0.44, 1.28)	0.289
G allele	1.25	(0.93, 1.68)	0.138	1.05	(0.80, 1.37)	0.729

MI: myocardial infarction.

**Table:** (b) Total mortality

	Dominant model			Recessive model		
	HR	(95% CI)	*P*	HR	(95% CI)	*P*
Age (10 years)	2.12	(1.89, 2.39)	<0.001	2.12	(1.89, 2.39)	<0.001
Male	1.26	(1.00, 1.60)	0.052	1.27	(1.00, 1.60)	0.051
Current smoking	1.01	(0.82, 1.24)	0.937	1.01	(0.82, 1.24)	0.941
Hypertension	0.91	(0.75, 1.09)	0.305	0.90	(0.75, 1.08)	0.271
Diabetes mellitus	1.55	(1.30, 1.84)	<0.001	1.54	(1.30, 1.84)	<0.001
Previous MI	1.19	(0.90, 1.57)	0.226	1.19	(0.90, 1.57)	0.230
Statins treatment	0.76	(0.51, 1.14)	0.188	0.77	(0.51, 1.15)	0.202
G allele	1.18	(0.95, 1.47)	0.135	1.11	(0.91, 1.35)	0.312

MI: myocardial infarction.
